# Fighting with Aging: The Secret for Keeping Health and Longevity of Naked Mole Rats

**DOI:** 10.14336/AD.2024.0109

**Published:** 2024-01-09

**Authors:** Wenjing Yang, Yifan Hu, Shufang Cui

**Affiliations:** ^1^Laboratory Animal Science Department, Basic Medical School, Naval Medical University, Shanghai, China.; ^2^Changhai Hospital, Naval Medical University, Shanghai, China; ^3^No. 904 Hospital of the PLA Joint Logistics Support Force, Wuxi, China

**Keywords:** naked mole rat, aging, ROS, genome, metabolism

## Abstract

Health and longevity are the dreams of mankind and the main field of the medical community. The naked mole rat (NMR) is a unique murine animal with extremely long lives (exceeding 38 years), revealing little signs of aging such as reproductive decline, neural degenerative diseases, and cancer. They provide us with valuable perspectives on preventing age-related diseases. This review systematically summarized the characters of different systems of naked mole rats in aging resistance, and furtherly exploited the mechanisms for aging resistance form genome, telomeres, protein recycling, metabolism, and oxidative stress attitudes. As a species with a high similarity with human beings, it cannot be ruled out that after reasonable validity, safety and ethical evaluation, the dominant genes of naked mole rats will be developed in medical transformation, to realize the dream of human health and longevity.

Longevity and health should be a major goal of all the people. The occurrence of chronic diseases is often positively correlated with aging [1], such as cancer, cardiovascular disease, and neurodegeneration [2-4]. Many studies have been performed in model animals such as Caenorhabditis elegants and lab mice, such as the cGas/Sting pathways and mTOR pathways [5, 6]. These animals are excellent in Drug evaluation and mechanism research, but show limitations in providing novel perspectives to fight age-related diseases from evolutionary ways. The naked mole rat (NMR) is a unique murine animal with extremely long lives (exceeding 38 years) [6], revealing little signs of aging such as reproductive decline [7], Alzheimer’s disease [8], and cancer [9], et al. Therefore, it is imperative to find effective gene, environmental, and drug interventions to delay aging, improve functional loss, and reduce the incidence of late-life disease. Advantages in omics and genetic editing by CRISPR/Cas9 system will enable the transmission of advantageous genes across species. This study will systematically summarize the physiological characteristics of naked mole rats, and exploit the mechanisms for keeping healthy and longevity, to provide a useful valuable message for all human beings’ dreams of health and longevity.

## 1. Characteristics of long-lived naked mole rats

The Naked mole rat, who lives in a resource-poor, humid, and harsh subterranean environment, is just one of more than 50 kinds of subterranean rodent species worldwide [10]. The life quotient of naked mole rats is (the ratio of maximum observed lifespan to weight-predicted lifespan) greater than 4, similar to human beings [11]. They are eusocial animals, like bats, social bees, wasps, and ants. They are resistant to cancer [12, 13] and hypoxia [14, 15]. Besides, there remains a large area to draw a systematic picture of their evolutional routes.

## 2. Physiological performances of aging resistance in naked mole rats

### 2.1 Hematopoietic and immune system characters in aging resistance

Immune senescence related to aging, is characterized by changes in immune components, such as loss of adaptive immune diversity [16], susceptibility to infection in older people, autoimmunity, and cancer. But Naked mole rats show delayed immune senescence [17]. Naked mole rat hematopoietic systems consist of a large pool of quiescent hematopoietic stem cells (HSCs). Transcriptomic data show molecules in cell protection pathways but not inflammatory markers are upregulated during aging in naked mole rats, which is quite different from human beings and lab mice. The inherent myeloid bias in the bone marrow of naked mole rats does not predispose hematopoietic stem cells and progenitor cells to reduced lymphocyte differentiation, thus naked mole rats do not reveal an age-related reduction in early T-cell progenitors [18]. So, there is no thymic involution in Naked mole rats until 11 years old [17]. The high levels of Aire and FOXN1 also ensure thymic function at neonatal levels [17]. Erythropoiesis in the spleen sustains into adulthood, which is also crucial for the long-term survival of naked mole rats [19]. Due to an increase in myeloid cells and a decrease in lymphoid lineages in spleens, Naked mole rats exhibit a lower level of adaptive immunity [19], but a high level of innate immunity [20], which may be energy-efficient in immune surveillance to defend heterologous infection. The Langerhans cells that settle in the skin (derived from bone marrow and spleen) are conserved and show no signs of aging in Naked mole rats [21], which may also play critical roles in protecting them from various infections. Interestingly, the number of skin stem cells and the epidermal gene levels showed surprising stability during aging [21], indicating the excellent renewal and healing power in naked mole rats [22]. Functional skin healing experiments have shown that wound closure rates in young and old animals are strictly equivalent to 33, exhibiting a very strong anti-aging phenotype. RNAseq analysis revealed that the transcription levels of several longevity-related genes (IGFBP3, IGF2BP3, Ing2) and tumor suppressor genes (BTG2, CDKN1A, CDKN2C, Dnmt3a, HIC1, SOCS3, SFRP1, SFRP5, THBS1, TSC1, ZFP36) were significantly increased in senescent NMR skin compared with aged human or mouse skin [22].

### 2.2 Forever young cardiovascular systems of naked mole rats

The naked mole rat hearts show little changes in aging hearts. There was no age-related change in NMR left ventricular function either at rest or under exercise stress. ECG analysis did not find any arrhythmias in all ages of naked mole rats, while it significantly increased in aged mice. The E/A ratio of only female naked mole rats (used to evaluate ventricular diastolic function) decreased significantly with age. Interestingly, a decrease in diastolic function in female naked mole rats does not lead to cardiovascular disease (CVD) and morbidity or mortality [23]. In particular, QRS, an important aging sign of heart function in lab mice, showed no incensement in both young and aged naked mole rats [23]. Morphologically, cardiomyocyte cross-sectional area left ventricular size, and left ventricular wall thickness in naked mole rats over their life expectancy [24], which are great differences between humans and lab mice [25]. Although cardiomyocyte hypertrophy has been found in naked mole rats, it does not develop into heart disease [23, 24, 26, 27]. In the study of cardiac fibrin profile analysis, naked mole rats mainly depend on β-MHC, which is economical in ATP consumption compared with α-MHC [26].

Age-related arteriosclerosis, aortic blood pressure, and pulse wave velocities have not been fund changed with aging in naked mole rats [24]. Though high levels of oxidative have been detected in arteries, there were quite a few lesions in vascular or apoptosis in vascular endothelial cells [28, 29]. Apoptotic cells were significantly increased (about 250-300%) in the arteries of aged rats (2 years old), 5-6-fold (about 50%) of those in aged naked mole rats (12 years old). This suggests that blood vessels in naked mole rats tolerate high levels of oxidative stress to maintain young vessel stabilities [28].

### 2.3 Aging resistance in naked mole rat central nervous system

The hypoxia tolerance of naked mole rats plays an important role in aging resistance. There are three ways to help naked mole rats switch between normoxia and hypoxia rapidly. First of all, it has been found a unique anaerobic glycolysis pathway that rapidly energizes nerve cells in naked mole rats during hypoxia [15]. Secondly, there are sustained greater proportion of the GluN2D subunit till adulthood compared with mice, which play critical roles in delaying calcium entry into neurons when hypoxia exposure [30]. Thirdly, reducing the energy expenditure of nerve cells. Though much more neural stem/progenitor cells (NS/PCs) in naked mole rats proliferated more slowly and stayed in the G0/G1 phase. But after stimulus such as γ irradiation, NMR-NS/PCs initiate inner mechanisms rapidly against DNA damage and keep cells away from dying [31]. Finally, there is postnatal neuromorphogenesis and spatial synaptic refinement in partial brain regions of naked mole rats, including the hippocampus and olfactory, helping naked mole rats adapt to extreme hypoxia and avoid neurodegenerative processes [32]. Interestingly, although Alzheimer's disease (AD)-associated proteins (Aß and phosphorylated tau) are extremely high in naked mole rat brains, neither Aß plaques, neurofibrillary tangle, nor neuronal loss occurs. This provides new insights into the mechanisms of AD [33, 34]. Maybe the low aggregation rate of Aβ_1-42_ in naked mole rats raises the threshold for neuronal tolerance toxicity [35]. Other proteins in the naked mole-rat brain are also of interest. It has been shown that NRG-1 prevents the impairment of hippocampal function by endogenous and exogenous neurotoxins [36]. Proteomic studies of the naked mole rat brain identified nine proteins associated with neurite growth and neurotransmission: Cofilin-1, dihydro-pyrimidinase-related protein 2 (isoform 2), aka collapsin response mediator protein 2, spectrin alpha chain (isoform 3), Septin-7, Syntaxin-binding protein 1, synapsin-2 isoform IIB, and dynamin1 (isoform 3 and 4). These proteins increase significantly with age and play critical roles in maintaining the neuroplasticity of naked mole rats during aging [37].

### 2.4 Unique musculoskeletal system

Although there were no significant differences between naked mole rats and other Girls in terms of bone composition, mechanical properties, growth patterns, and basic structure, the cortical bones of the naked mole rats are completely circumferential and layered, lacking a mineralized cartilage island. Furthermore, the naked mole rat skeletons reveal no evidence of remodeling at any age [38]. The skeletal homeostasis, bone mineral structure, and mechanical properties are stabilized in aged naked mole rats, but not in aged mice and human beings [25]. High levels of high molecular weight hyaluronic acid (HMWHA) in naked mole rats may be responsible for the resistance to trauma and common degenerative diseases such as osteoarthritis [39].

Healthy skeletal muscle tissue persists in naked mole rats for decades. Muscle fiber integrity and mitochondrial ultrastructure were well maintained in aged naked mole rats, revealing few signs of age-related muscle atrophy or mitochondrial dysfunction. Mitochondrial complex IV expression and activity remained stable, but complex I expression was significantly reduced in naked mole rats. Mitochondrial DNA copy number increased significantly in skeletal muscle fibers of naked mole rats with age [40], which may be beneficial for maintaining normal movements of muscles. Interestingly, mitochondrial DNA rearrangements only exist in senescent skeletal muscle tissue in naked mole rats, but not in human beings and other rodents. These changes may be responsible for the physical function of naked mole rats to remain healthy throughout their lives.

### 2.5 Sustained female reproduction ability

Although reproduction ensures the survival of the entire species, it reduces an individual's lifespan [41]. This theory is not true in the queen of naked mole rats. The Queen of naked mole rats, which breed throughout their lifespans, does not suffer from the energy costs of reproduction. Maybe extremely high levels of RRAGB and TMEM8C are contributed to the longevity and sustained reproduction ability [41, 42]. As eusocial animals, the queen has absolute power to dispatch energy and ensure the survival of the community, which is an important way for this species to gain a foothold on Earth. This is the result of a eusocial lifestyle with the most arranged energy at the expense of the reproductive capacity of the workers.

On the other hand, the reproductive senescence of female naked mole rats is also manifested in expanded ovarian reserves [43]. In human beings and mice, a limited ovarian reserve is established from birth [44], and reproductive capacity gradually declines as the reduction of ovarian reserve with age [45, 46]. The fertility of the mice was maintained for a maximum of 9 months [47]. The naked mole-rats ovaries remained largely germ cell at 90 days after birth and showed a 10-fold greater ovarian reserve by 6 months than lab mice [48]. This is one of the main reasons for the queen in the colony to restore her reproductive ability until the end of her life [48].

## 3. Mechanisms of aging resistance in naked mole rats

This evolutionary tinkering, and the consequent alteration or recycling of various components, promotes integrative adaptation through a process of natural selection [49]. Large differences in the maximum lifespan potential of species [MLSP] must ultimately be encoded by genes. However, if a specific lifespan program exists, a genetic response mutant can identify such a program and help human beings achieve immortality.

Large amounts of genomic and transcriptomic data from naked mole rats have been processed in recent studies [13, 15, 50]. About 88% of the naked mole rat genes are orthologous to those of human [50].

In comparison with mammals, there are 750 acquired and 320 lost genes found in naked mole rats [50], and about 66 unique additional genes have been found in naked mole rats [50]. Naked mole rats strive to maintain genetic stability, and on the other hand, make genetic changes at minimal cost to adapt to extreme environments, making their evolutionary differential genes of great value to human beings.

## 3.1 Roles of the genome in aging resistance

### 3.1.1 Gene evolutions

Gene evolutions make naked mole rats inhabit the environment. Interestingly, naked mole rats are genetically much similar to human beings than mice [51]. Interspecies differences in mutation rates are generally considered to indicate different evolutionary pressures. Compared with mice and guinea pigs, the incidence of synonymous and non-synonymous differences in naked mole rats decreased significantly [51].

Activities of splicing factor are higher in naked mole rats from youth to old [52], which may contribute to better molecular stress responses and avoidance of senescence [52]. Comparative analysis revealed that ADAMTS9, the known inhibitor of the mTOR pathway, performing a driver of senescence, is extremely high in bats naked mole rats at all ages [53]. The convergent evolution of ADAMTS9 may therefore be responsible for the extraordinary longevity and anti-tumor activity of bats and naked mole rats. Elevated levels of p16 are associated with the early contact inhibition in dermis fibroblasts of naked mole rats [54], due to the unique splicing pattern of pALT^INK4a/b^ in the Ink4 transposon of naked mole rats, thus affecting cell cycle progression and tumorigenesis [55, 56].

There was no significant differences in copy number between the naked mole rat genome and that of human oncogenes and tumor suppressor gene [55]. Moreover, expression levels of many enzymes targeting DNA repair are higher in the liver, brain, and testis than in mice [55, 56], including enzymes involved in tumor suppression (e.g. TP53), base excision repair, mismatch repair, and non-homologous end ligation [56].

### 3.1.2 Maintenance of Genome Stability

DNA damages affect most aspects of the aging phenotype [57]. There was not only lower mutation frequency but also stronger DNA repair in naked mole rats, such as higher levels of Nrf2 activity in naked mole rats than mice [58]. RNA-seq data on the brains, livers, and kidneys of newborn, young (4-year-old), and old (20-year-old) naked mole rats showed that few genes in naked mole rats show differential expression between 4 to 20 years old, especially in the brain. For example, genes associated with macromolecular degradation, such as GSTA1, DERL1, and GNS, are not upregulated with age [59]. Genes encoding mitochondrial proteins (NDUFB11, ATP5G3, and UQCRQ) [59] are not downregulated, which is consistent with the stable maintenance of mitochondrial function during aging. There are also higher copy numbers of CEBPG (a DNA repair regulator), Tinf2 (a protector of telomere integrity), and α-2 macroglobulin (A2M) [60] in the naked mole-rat liver, which play an important role in supporting gene stability.

Moreover, naked mole rat fibroblasts are resistant to radiation-induced apoptosis. It takes a much higher dose of γ-ray irradiation to trigger naked mole rat cell senescence [61]. Gene expression analysis of senescence-related changes showed that senescence-related secretory phenotype genes in naked mole rat cells were similar to mice, including induction of SASP, IFN, TNF, inhibition of protein translation, and cell cycle. The insufficient protection mechanisms in DNA metabolism, transcription, and translation [61], make mice susceptible to induced senescence.

### 3.1.3 Gene expression and regulation

Interestingly, naked mole rats reveal senescence in epigenetic levels. Lnc RNAs in naked mole rats share many similarities with other vertebrate species, such as tissue specificity and low expression. However, the shortest Lnc RNA has only been found in naked mole rats, compared to other long-lived species such as Bowhead whales and Brandt's bat [62]. Besides, CpG subpopulations underwent similar methylation changes during aging in naked mole rats compared with human beings [63].

### 3.2 Ambiguous roles of telomeres in aging resistance

In most eukaryotes, telomere shortening is thought to be critical in senescence. When they reach a critical length the cell enters a state of senescence, which is termed the 'senescence/senescence replication theory'. However, long telomeres and high telomerase activity are tumor-promoting factors. The largest comparative study of telomeres and telomerase involving more than 60 mammalian species has found that smaller, shorter-lived species tend to have longer telomeres and higher levels of telomerase [64]. In summary, the evolution of macrosomia and longevities appears to be closely related to the evolution of short telomeres and telomerase inhibition, possibly to suppress tumors.

Telomeres of naked mole rats are similar in length to those of human beings, and much smaller (1/3 to 1/2) than those of mice and rats [64]. Interestingly, the relative telomere length (RTL) in rat tissues (kidney, lung, and muscle) decreases with age but shows a slightly significant elongation in naked mole rats with age oppositely [65, 66]. This phenomenon may be caused by low telomerase activity in naked mole rats, which is demonstrated by the tight binding of TRF1 to telomeres in naked mole rats, leading to lower cancer incidence and longevity [67]. Besides, TERT showed stable expression at all ages, also consistent with the role of the telomerase complex highlighted by positive selection on TEP1 and TERF1 [50]. Currently, the role of telomeres in aging remains unclear, and naked mole rats may provide novel attitudes exploring relations between telomeres and aging.

### 3.3 Protein recycling and aging resistance

The cellular proteome performs highly diverse functions to sustain life [68]. It has involved a complex network of protein quality control (PQC) pathways in cells, including degradation pathways and numerous chaperones and co-chaperones, to assess protein quality, and by refolding or eliminating proteins through degradation pathways to initiate appropriate responses to mitigate damage. But this quality control system will decline as cell senescence, leading to a series of diseases, including neuro-degeneration and so on.

In general, the lifespan is negatively correlated with the turnover of high-abundance proteins. Compared with mice, cells from naked mole rats exhibit slower protein turnover and a robust protein homeostasis system [69], which reduces the risk of many unnecessary mutations. The unique 28S rRNA in naked mole rats guarantees high fidelity and stability in protein synthesis [70]. It is shown that proteolytic degradation mechanisms and maintenance of protein quality play a key role in the lifespan of rodents [71]. An efficient ubiquitin-proteasome system (UPS) in naked mole rats enables the rapid degrading of misfolded proteins [72]. During senescence, higher proteasome activities and autophagic rate have been found in naked mole rat organs than those of mice [69]. High levels of the key chaperones HSP72, HSP40, and HSP25 in naked mole rats protect proteasome function from cellular stressors [73]. There was a significant positive correlation between MLSP, HSP25, HSF1, proteasome activity, and autophagy-related protein 12 (ATG12). High basal autophagic activity in naked mole rat cells mediated by ATG5 contributes to the inhibition of p53/Rb-induced apoptosis and increases their lifespan [74].

**Figure 1.**
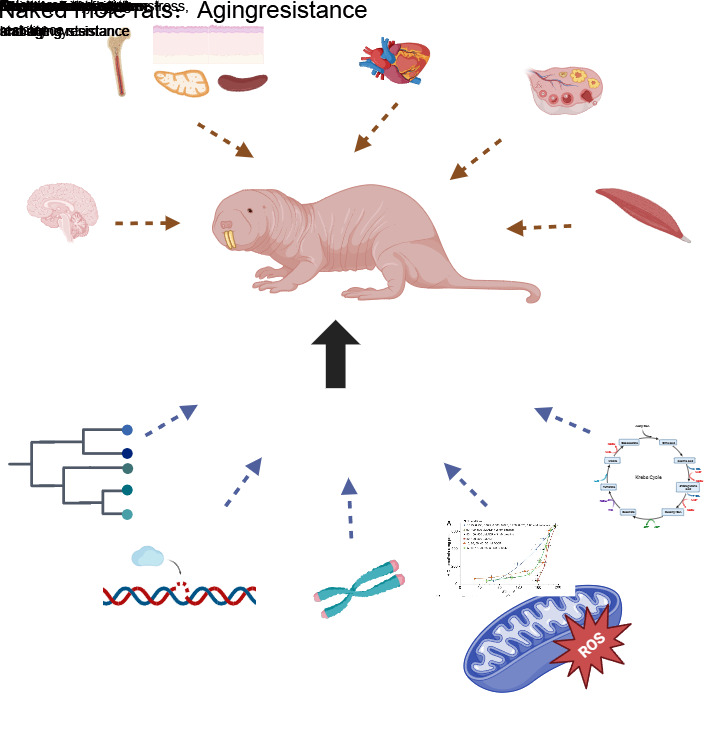
**Characters and mechanisms of aging resistance in naked mole rats**. Various organisms of naked mole rats show aging resistance. The underlying mechanisms may be due to gene evolutions, maintenance of genome stability, telomeres, ros, and protein recycling mechanisms.

### 3.4 Metabolism and aging resistance

A moderate reduction of O_2_ concentration may be beneficial to longevity [50, 75]. The overall metabolic rate was maintained at a lower level throughout the whole life span in naked mole rats [76]. The stable levels of HIF-1α ensured the stability of naked mole mouse proteins at different oxygen concentrations, increasing the efficiency of oxygen consumption. The attenuation of the HIF-1α function is related to senescence of mice [77]. Besides, the use of fructose to produce ATP under special conditions is also a relatively energy-saving method [15]. Interestingly, the gut microbiota of naked mole rats is capable of using soil sulfate as a terminal electron acceptor to maintain anaerobic oxidative metabolism [78]. Amino acid metabolism is more dominant than fatty acid metabolism in naked mole rats [79, 80]. In terms of protein metabolism, both PK levels and PKM levels in naked mole rats increased with aging, accompanied by a decreased phosphorylation state. High PKM activity is responsible for raising the efficiencies of cellular function in aging.

### 3.5 Controversial theories on oxidative stress and aging

Oxidative stress theory predicts that the rate of oxidative damage generation and accumulation in long-lived species should be lower than in short-lived species, and these differences should be evident even when comparing younger, healthy individuals [81]. Multiple comparative studies have shown that mitochondrial ROS (mtROS) production rates and oxidative damage to mitochondrial DNA are inversely associated with maximal lifespan, especially in long-lived animals [82]. But the long-lived naked mole rat is an exception. The horizontal comparison showed that the level of oxidative stress damage accumulated in the naked mole rats was much higher than that of mice of comparable age, but the longitudinal comparison showed that the elderly naked mole rats reveal similar high levels of oxidative stress damage as the young naked mole rats [83]. This is a challenge to the classical theory of oxidative stress aging and may force a re-evaluation of the interrelationship between oxidative stress damage and aging (Fig 1.).

## 4. Conclusions

Health and longevity are also the dreams of mankind and the main field of the medical community. For a long time, evolution, long-lived Naked mole rats have developed an efficient way of using energy, including their true social lifestyle, as well as the way the organism metabolizes and distributes energy. Adjustments at the genome and protein levels allow the species to exist for generations. With the update of gene editing, gene therapy, and cell therapy technology, some researchers have verified the function of the dominant genes of naked mole rats on other species and effectively improved some pathological states of other species [9]. But quite a lot of discoveries are demonstrated by omics, without experimental verification, which may be a restriction in medical transplantation (Table 1). But, as a species with high similarities with human beings, it cannot be ruled out that after reasonable validity, safety, and ethical evaluation, the dominant genes of naked mole rats will be developed in medical transformation, to realize the dream of human health and longevity.

**Table 1:** Advantages and limitations in applications of naked mole rats.

Advantages in applications	Limitations in applications
Unique genes/proteins in aging resistance: pALT^INK4a/b^ [54], HMWHA [9], 28S rRNA [70]	Roles of telomeres in aging resistance [50, 64-67]
Common genes with different expression levels: ADAMTS9 [53], Nrf2 [58], GSTA1 [59], DERL1 [59], GNS [59], CEBPG [60], Tinf2 (a protector of telomere integrity) [60], A2M (α-2 macroglobulin) [60]	Oxidative stress and aging [82, 83]
Epigenetics: Lnc RNAs [62], CpG subpopulations [63]	
Protein recycling and aging resistance: HSP72 [73], HSP40 [73], HSP25 [73], ATG5 [74]	
Metabolism: stable HIF-1α [77], PK and PKM levels [79, 80]	
